# Evaluating the Role of Retrieval Bags in Mitigating Contamination During Minimally Invasive Colorectal Surgery

**DOI:** 10.3390/jcm15020726

**Published:** 2026-01-15

**Authors:** Javier Valdes-Hernandez, Andrea Balla, Christof Mittermair, Christian Obrist, Juan Carlos Gómez-Rosado, Katharina Pimpl, Eberhard Brunner, Jan Schirnhofer, Helmut Weiss, Salvador Morales-Conde

**Affiliations:** 1 Department of General and Digestive Surgery, University Hospital Virgen Macarena, University of Sevilla, 41009 Sevilla, Spain; 2 Unit of General and Digestive Surgery, Hospital Quirónsalud Sagrado Corazón, 41013 Sevilla, Spain; 3 Department of General and Visceral Surgery, Saint John of God Hospital, Teaching Hospital of the Paracelsus Medical University Salzburg, Kajetanerplatz 1, 5010 Salzburg, Austria

**Keywords:** minimally invasive colorectal surgery, specimen retrieval bag, bacterial contamination, tumour cell spillage, cytological analysis

## Abstract

**Objective:** To evaluate the extent of intraoperative bacterial and tumour cell spillage during minimally invasive colorectal surgery and to assess the protective value of systematic specimen retrieval using a tear-proof extraction bag. **Methods:** This multicentre, prospective observational study included patients undergoing conventional or single-port laparoscopic colorectal surgery for adenocarcinoma, premalignant polyps, or chronic diverticulitis. Three intraoperative samples were obtained for microbiological and cytological analysis: after pneumoperitoneum induction (sample 1), after vascular ligation and bowel division (sample 2), and after specimen extraction using a retrieval bag (sample 3). **Results:** Eighty-eight patients were included. Bacterial contamination increased significantly throughout the procedure occurring in 11.4% of sample 1, 37.5% of sample 2, and 67% of sample 3 (*p* < 0.001). When sample 1 was positive, sample 2 was positive in 100% of cases; when sample 2 was positive, sample 3 was positive in 79% of cases. In 33 patients (37.5%), bacterial growth was detected exclusively in sample 3. Contamination in sample 2 was significantly associated with surgical approach (*p* = 0.013), anastomotic technique (*p* = 0.022), and malignant disease (*p* = 0.038). A longer hospital stay was significantly associated with contamination in samples 1 and 2 (*p* = 0.014 and *p* < 0.001, respectively). No tumour cells were detected in any sample, except for one case showing atypical cells without clinical relevance in sample 3. **Conclusions:** Intraoperative bacterial contamination progressively increases during minimally invasive colorectal surgery, peaking after specimen extraction. Most clinical and surgical variables did not significantly influence contamination rates. The use of a specimen retrieval bag demonstrated a potential protective effect by containing bacterial spillage. However, no protective effect regarding tumour cell dissemination could be demonstrated based on cytology analysis.

## 1. Introduction

Despite the well-established advantages of minimally invasive surgery (MIS) in colorectal procedures compared to open surgery, particularly in terms of perioperative outcomes and its demonstrated non-inferiority in oncological results, comprehensive studies evaluating intraoperative spillage of bacteria and tumour cells into the peritoneal cavity, as well as at port sites and laparotomy incisions for specimen extraction, remained limited [1,2,3,4,5,6,7,8]. Such spillage is most commonly associated with tumour manipulation or specimen retrieval [7,8]. Concerns regarding this phenomenon have increased with widespread adoption of MIS, as the creation of pneumoperitoneum and the need for specimen extraction through small incisions may potentially increase the risk of peritoneal or port-site metastasis [7].

To mitigate these risks, several preventive strategies have been implemented [3,8,9,10,11], including the use of no-touch techniques during oncologic resections, and the routine application of wound protectors or specimen retrieval bags to minimize contamination [8]. As a result of these measures, the port-site recurrence incidence has markedly declined, decreasing from approximately 21% in the early 1990s, when laparoscopic colorectal surgery was first introduced, to around 3% in more recent years [3,9,10,11].

Although precise dissection and specimen resection can be effectively achieved laparoscopically, additional measures, including the use of a wound protectors, peritoneal irrigation, and evacuation of insufflation gas prior to trocar removal, are considered essential to reduce intra-abdominal contamination [12,13].

Peritoneal seeding and tumour recurrence remain recognized complications of laparoscopic colorectal surgery and may compromise the curative potential of the procedure [8]. While mechanical compression of the tumour during handling has been proposed as a simple mechanism for cell dissemination, the underlying processes appear to be more complex [8]. Several hypotheses suggest that increased intra-abdominal pressure, the physical characteristics of the insufflation gas, and temperature fluctuations may impair peritoneal integrity and create an environment that favours tumour cell implantation [8].

During specimen extraction through small incisions, as commonly performed in MIS colorectal surgery, the mechanical tension applied to the specimen may lead to the inadvertent release of bacteria or viable tumour cells into the peritoneal cavity [7], thereby increasing the risk of postoperative infection or tumour recurrence [7]. The routine use of tension-resistant specimen retrieval bags during MIS procedures may help to mitigate this risk.

The aim of the present study is to quantify the extent of intra-abdominal bacterial and tumour cell spillage during specimen retrieval in MIS colorectal surgery and to evaluate the potential protective effect of specimen retrieval bags.

## 2. Material and Methods

This is a multicentre prospective observational study. Institutional review board approval (0501-N-15) and informed consent from all participants were obtained.

### 2.1. Patient Selection

Patients who underwent conventional or single port laparoscopic colorectal surgery, from September 2013 to February 2015 in three centres University Hospital “Virgen Macarena”, Sevilla, Spain, University Hospital “Virgen del Rocio”, Sevilla, Spain and Saint John of God Hospital, Salzburg, Austria, were included in the study.

### 2.2. Inclusion and Exclusion Criteria

Patients aged 18 years or older with colorectal adenocarcinoma, giant polyps with high-grade dysplasia not amenable to endoscopic resection, or chronic diverticulitis who underwent surgery in elective setting were included in the analysis. Patients with contraindications to the laparoscopic approach, those undergoing emergency setting, patients who received neoadjuvant radio–chemotherapy, those with stage cT4, or patients with diverticular abscess or active infection within one month prior to surgery were excluded.

### 2.3. Study Outcomes

The primary outcome was the assessment of intraoperative bacterial growth, and the detection of atypical endothelial cells retrieved from the specimen retrieval bag. The secondary outcome was the evaluation of recurrence rate at one year follow up.

### 2.4. Surgical Techniques

Surgery was performed according to the underlying colorectal disease, and all participating centres adhered to an identical standardized study protocol encompassing surgical techniques, perioperative management and aseptic measures, specimen extraction methods, and intraoperative sample collection and microbiological processing, in accordance with shared operative and study protocols. Patients with adenocarcinoma or giant polyps not suitable for endoscopic resection underwent oncological resections (right or left hemicolectomy, or anterior resection of the rectum), while patients with chronic diverticulitis underwent sigmoid resection.

For each patient, the surgeon chose, at their discretion, whether to perform a conventional or single-port laparoscopic approach. In patients undergoing conventional laparoscopic surgery, pneumoperitoneum was established using a Veress needle at Palmer’s point, whereas in single-port procedures, pneumoperitoneum was created directly at the single-port insertion site.

In all procedures, specimen retrieval was performed using a tear-proof bag (Endobag™ Specimen Retrieval 15 mm, Covidien, Mansfield, MA, USA) together with a wound protector (Alexis, Applied Medical, Rancho Santa Margarita, CA, USA).

In single-port surgery, the device (GelPOINT™, Applied Medical, Rancho Santa Margarita, CA, USA; SILS™ Port, Covidien, Mansfield, MA, USA; OCTO-Port^®^, AFS Medical, Teesdorf, Austria) was selected based on the patient’s characteristics or the surgical procedure, and placed mainly at the umbilicus, at the site of a possible protective ileostomy (in anterior resection of the rectum), or at the site of a pre-existing scar.

In laparoscopic right hemicolectomy, the intracorporeal mechanic anastomosis is performed with a linear stapler with 60 mm purple cartridge [1]. The enterotomy is then closed using two continuous sutures with absorbable 2.0 barbed suture [1]. The decision to perform an intracorporeal or an extracorporeal mechanical anastomosis was made by the surgeon [14]. The specimen was extracted through a Pfannensteil incision in case of intracorporeal anastomosis or through an enlargement of the subcostal trocar orifice in case of extracorporeal anastomosis, whereas in single-port right hemicolectomy, it was extracted through the single-port incision.

For the purposes of the study, in left hemicolectomy, sigmoidectomy, or anterior resection of the rectum, the specimen was extracted via Endo bag after proximal and distal division, through either a Pfannenstiel incision or the single-port incision. Anastomosis was fashioned using the double-stapling technique [15].

### 2.5. Sampling for Bacteriological and Cytological Analysis

Twenty millilitres of normal saline were instilled into the right parietocolic gutter for right-side colonic lesions, into the Morrison’s pouch for transverse colonic lesions, and along the left parietocolic gutter for left-sided, sigmoid, or rectal lesion, and were subsequently aspirated for analysis.

For each patient, three samples were collected and analyzed at predefined stages of the surgical procedure. The first sample was obtained after pneumoperitoneum induction and initial abdominal exploration. The second sample was collected after vascular ligation and bowel division, using the same technique described above. For the third sample, following extraction of the resected bowel within Endo bag, 20 mL of normal saline were instilled into the retrieval bag and subsequently aspirated for analysis.

All samples underwent bacteriological analysis, including aerobic and anaerobic bacteria, as well as cytological examination for each patient.

### 2.6. Study Design

Gender, age, body mass index (BMI), comorbidities, American Society of Anesthesiologists (ASA) grade, previous abdominal surgery, indication for surgery (adenocarcinoma, giant polyps with high-grade dysplasia), lesion site (right colon, transverse colon, left colon, sigmoid colon, recto-sigmoid junction, rectum), approach (conventional or single port laparoscopy), surgical procedure (right and left hemicolectomy, sigmoid resection and anterior resection of the rectum), intraoperative complications, conversions to open surgery, type of anastomosis (intracorporeal side-to-side, intracorporeal end-to-end, extracorporeal side-to-side, extracorporeal side-to-end), operative time, 30-day postoperative complications (graded according to the Clavien-Dindo classification [16]), length of hospital stay (LOS), mortality, staging, number of harvested lymph nodes, resection margin, recurrence rate at one year follow up, microbiological and cytological data obtained from the three intraoperative samples were recorded in Microsoft Access programme (Microsoft Corporation, Redmond, WA, USA).

### 2.7. Statistical Analysis

Continuous variables are expressed as mean ± standard deviation, and categorical variables as absolute frequencies and percentages. Depending on the distribution of the continuous variables, comparisons between groups were performed using either Student’s *t*-test for normally distributed data or the non-parametric Mann–Whitney U test when normality could not be assumed. Associations between categorical variables were evaluated using the Chi-square test. These statistical tests were selected to appropriately account for the scale of measurement and the underlying distribution of each variable. A *p* value lower than 0.05 was considered statistically significant. All analyses were performed using SPSS software version 22.0 (SPSS Inc., Chicago, IL, USA).

## 3. Results

A total of 88 patients were included in the analysis. The majority were male (62.5%), with a mean age of 65.4 ± 11.5 years and a mean BMI of 26.7 ± 4.2 kg/m^2^. According to the ASA classification, 15 (17%), 42 (47.7%) and 31 (35.2%) patients were graded ASAI, II and ASA III, respectively. Thirty-one (35.2%) patients underwent previous abdominal surgery (Table 1).

Fifty-one (58%) patients underwent surgery for colorectal adenocarcinoma, 18 (20.5%) for giant polyps with high-grade dysplasia, and 19 (21.6%) for chronic diverticulitis. The most frequent lesion sites were the right colon (33, patients 37.5%) and sigmoid colon (29 patients, 33%) (Table 1).

Perioperative outcomes are summarized in Table 2. A single-port laparoscopic approach was used in 49 patients (55.7%) while the remaining 39 patients (44.3%) underwent conventional laparoscopy. The most commonly performed procedures were right hemicolectomy (36 patients, 40.9%), sigmoidectomy (23 patients, 26.1%), anterior resection of the rectum (16 patients, 18.2%), and left hemicolectomy (13 patients, 14.8%). Intraoperative complications occurred in four (4.5%) patients (three bleeding and one positive hydropneumatics test). Conversions to open surgery did not occur.

Mechanical anastomotic techniques varied across the cohort. Intracorporeal side-to-side and intracorporeal end-to-end anastomoses were each performed in 25 patients (28.4%), while extracorporeal side-to-side and extracorporeal side-to-end anastomoses were performed in 20 (22.7%) and 17 (19.3%) patients, respectively. In one patient (1.1%), who underwent anterior resection of the rectum, a colostomy was fashioned instead of an anastomosis. Overall, the mean operative time was 128.1 ± 39.2 min.

Postoperative complications occurred in 24 patients (27.3%). These included ileus in 2 (2.3%) patients (one after right hemicolectomy and one after anterior resection of the rectum, Clavien-Dindo I); wound infections in 8 (1.8%) patients (seven after right hemicolectomy and one after anterior resection of the rectum, Clavien-Dindo II); pneumonia in 2 (2.3%) patients (one after right hemicolectomy and one after sigmoidectomy, Clavien-Dindo II); postoperative bleeding in 2 (2.3%) patients (both following right hemicolectomy, Clavien-Dindo III-b); and anastomotic leakage in 10 (11.4%) patients (four after anterior resection of the rectum, three after right hemicolectomy, and three after sigmoidectomy, Clavien-Dindo III-b).

The mean postoperative hospital stay was 8.4 ± 5.9 days, and mortality was nil.

Definitive histology revealed adenocarcinoma in 61 (69.3%) patients, no pathological lesions in 5 (5.7%) patients and diverticular disease in 22 (25%) patients. The mean number of harvested lymph nodes was 15.8 ± 6.1. No recurrence was documented at one year follow-up.

Microbiological findings are reported in Table 3. The analysis demonstrated a progressive increase in bacterial contamination across the three intraoperative sampling time points. In the first sample, which was collected after pneumoperitoneum induction, bacterial contamination was minimal. *Escherichia coli* was detected in 8% of patients, followed by *Enterococcus* in 2.3% with isolated cases of *Bacteroides vulgatus* and mixed bacterial flora observed in 1.1% of patients each (Table 3).

In the second intraoperative sample, collected after vascular ligation and bowel division, a higher rate of bacterial contamination was observed. *Escherichia coli* was detected in 18.2% of patients, mixed bacterial flora in 14.8%, and *Enterococcus* faecium in 5.7%. Other microorganisms, including *Clostridium*, *Bacillus magaterium*, Bacteroides, *Klebsiella pneumoniae*, *Bacteroides vulgatus*, *Lactobacillus*, *Morganella morganii*, *Prevotella denticola*, *Candida albicans*, and *Aeromonas hydrophila*, were identified in isolated cases, with individual frequencies ranging from 1.1% to 2.3% (Table 3).

In the third intraoperative sample, collected after specimen extraction using a retrieval bag, demonstrated the highest rate of bacterial contamination. *Escherichia coli* was identified in 29.5% of patients, mixed flora in 15.9%, and Bacteroides species in 12.5%. Additional microorganisms included *Enterococcus* (6.8%), *Streptococcus* (5.7%), *Bacteroides vulgatus*, *Pseudomonas aeruginosa*, and *Serratia marcescens* (each 5.7%), *Citrobacter* and *Morganella morganii* (each 2.4%), as well as *Candida*, *Enterobacter*, *Klebsiella pneumoniae*, *Parabacteroides*, and *Staphylococcus aureus* (each 1.1%) (Table 3).

Bacterial contamination was detected in 10 (11.4%) patients in the first sample, 33 (37.5%) patients in the second sample, and 59 (67%) patients in the third sample (*p* < 0.001 for all comparison). Overall, these findings demonstrate a progressive increase in intraoperative bacterial contamination, peaking after specimen retrieval, and are consistent with the degree of bowel manipulation and extraction.

The probability of obtaining a positive culture in the second sample when the first sample was already positive was 100%. Similarly, the probability of a positive culture in the third sample when the second sample was positive was 79%. Only seven patients had a negative third sample despite a positive second sample. In contrast, in 33 (37.5%) patients, bacterial growth was detected exclusively in the third sample, with no microorganisms identified in samples 1 or 2.

Table 4 reports the associations between patient characteristics, perioperative variables, and the presence of bacterial contamination across the three intraoperative samples. Most variables did not show statistically significant differences among the groups. Specifically, age, gender, BMI, ASA grade, surgical approach, anastomotic technique, postoperative complications (including wound infection and anastomotic leakage), and the presence of malignant disease were not significantly associated with positivity in 1 or sample 3. However, several significant associations were identified. The surgical approach was significantly associated with positivity in sample 2 (*p* = 0.013), suggesting that the type of laparoscopic access may influence contamination following vascular ligation and bowel division. Similarly, the anastomotic technique showed a significant association with sample 2 positivity (*p* = 0.022). The presence of malignant disease was also significantly correlated with positivity in sample 2 (*p* = 0.038), indicating a higher likelihood of bacterial contamination during oncologic resections at this operative stage.

LOS differed significantly in relation to positivity in samples 1 and 2 (*p* = 0.014 and *p* < 0.001, respectively), indicating that patients with bacterial contamination detected at earlier stages surgery tended to experience longer postoperative hospitalization. No significant association was observed between LOS and positivity in sample 3. Overall, these findings suggest that bacterial contamination occurring earlier during surgery (samples 1 and 2) is more closely associated with patient and procedure-related variables as well as postoperative outcomes, whereas contamination detected after specimen extraction (sample 3) appears to be less influenced by these factors.

Cytological analysis was performed in all 69 patients who underwent surgery for neoplastic or premalignant disease. No tumour cells were detected in any of the three samples. Only one case demonstrated the presence of atypical cells on cytological examination. These findings occurred in a patient who underwent single-port right hemicolectomy for adenocarcinoma with an intracorporeal side-to-side anastomosis. This patient had a T3N0 right colon adenocarcinoma, and postoperative complications did not occur.

## 4. Discussion

This study demonstrates that intraoperative bacterial contamination during minimally invasive colorectal surgery is a progressive phenomenon that peaks at the moment of specimen extraction, highlighting a critical step in the procedure. Despite minimal contamination at the beginning of the operation, more than two-thirds of patients exhibited bacterial growth in the sample obtained from the retrieval bag, underscoring the relevance of this protective device. Importantly, most patient- and procedure-related variables did not significantly influence contamination in the final sample, suggesting that spillage is largely inherent to specimen manipulation rather than to surgical technique or clinical characteristics. The identification of atypical cells in only one patient, without postoperative clinical consequences, further indicates that the risk of tumour cell dissemination is extremely low. Overall, these findings reinforce the pivotal role of specimen retrieval bags in limiting intra-abdominal contamination and supporting oncological safety in minimally invasive colorectal surgery.

Surgical site infection (SSI) remains one of the most frequent postoperative complications, affecting approximately 5% of all surgical patients and up to 30–40% of those undergoing abdominal procedures, depending on the degree of contamination [17,18,19]. These infections significantly increase morbidity, mortality, and healthcare expenditure [19]. In the United Kingdom, for example, SSIs have been shown to almost double the length of hospital stay, with additional costs per patient varying widely according to the procedure and severity of infection [20,21,22]. Because most SSIs originate from endogenous microbial contamination of the wound, the surgical community has repeatedly explored strategies that provide a physical barrier to protect incision sites, highlighting the longstanding interest in methods that minimize bacterial exposure during operative procedures [19]. For this reason, protection of the surgical wound during laparoscopic oncological surgery has been recommended since the early adoption of this approach for colonic cancer [19]. This maneuver is therefore considered a fundamental step of minimally invasive oncological surgery [19].

During our initial experience with single-port colorectal surgery, we observed a notably increased rate of wound-related infectious complications, even though a wound protector was routinely employed [14]. This increase in morbidity, compared with other procedures performed through a transumbilical single-port approach, prompted us to adopt the systematic use of retrieval bags for specimen extraction. Moreover, to investigate a possible association between wound complications and intraoperative contamination, we began collecting and analyzing the fluid retrieved from the retrieval bag after specimen removal. This approach aimed to determine whether bacterial spillage during extraction could partially explain the higher incidence of wound infections observed in single-port colonic surgery.

Intra-abdominal infectious complications may arise from several high-risk steps intrinsic to colorectal procedures [23,24,25]. Heavy manipulation of the bowel, specimen transection, extraction through a restricted incision, and anastomosis creation, particularly when performed intracorporeally, have all been implicated as potential sources of bacterial translocation, as demonstrated in this study. It has been well demonstrated that laparoscopic surgery significantly reduces the incidence of surgical site infections compared with open procedures, and that the use of wound protectors further decreases the risk of contamination [23,24,25]. However, the mechanisms underlying the reported higher rates of postoperative intra-abdominal abscess in minimally invasive colorectal surgery have remained less clear. The present study provides novel insight into this issue by identifying the specific intraoperative phases most prone to contamination. The highest rate of bacterial positivity was found in the sample collected immediately after vascular ligation and bowel division, indicating that this step represents a critical moment for intra-abdominal bacterial spillage. This observation offers a plausible explanation for the occurrence of deep infectious complications despite the recognized benefits of laparoscopy on wound-related morbidity and underscores the importance of targeted preventive strategies during this phase of the operation.

In our study, a second peak of bacterial contamination was observed in the third sample, collected after specimen extraction. However, because a retrieval bag was systematically used in all procedures, the bacteria identified in this sample originated exclusively from the fluid contained within the specimen retrieval bag and did not result in contamination of the peritoneal cavity. This contrasts with situations in which the specimen is removed through a wound protector alone, without the use of a retrieval bag, where any bacterial load present in the final sample would be released directly into the abdominal cavity, an effect that becomes even more pronounced when specimen compression or squeezing is required to facilitate extraction through a small incision. The routine use of the retrieval bag in our series therefore provides a plausible explanation for why the marked increase in contamination observed in the third sample did not translate into an increased rate of wound infections. These findings strongly suggest that consistent use of a retrieval bag may play a protective role in preventing intra-abdominal contamination during specimen extraction, particularly in minimally invasive colorectal surgery where traction and compression of the specimen are frequently required.

Regarding intraperitoneal malignant cell seeding and the subsequent development of recurrence during laparoscopic oncologic surgery, it has been well established that the incidence is extremely low, a finding that is also supported by the results of the present study [8]. One of the main rationale for conducting this analysis was to determine whether tumour cell dissemination might occur during specimen manipulation and extraction; however, no objective evidence of malignant cell contamination was detected in any of the samples. It is likely that strict adherence to oncologically sound principles, particularly the no-touch technique, played a decisive role in preventing tumour cell spillage, thereby contributing to the absence of cytological positivity and early recurrence in our cohort.

This study presents several limitations that should be considered. First, its observational design does not allow for establishing causal relationships between intraoperative contamination and postoperative infectious outcomes. Second, the sample size, although adequate for describing microbiological contamination patterns, may be insufficient to detect rare events such as tumour cell dissemination or uncommon complications, particularly in the context of cytological analysis. In this regard, the limited number of patients undergoing cytological assessment may have reduced the ability to identify minimal tumour cell spillage. Moreover, cytological evaluation relied on standard microscopic techniques, which may lack sensitivity for detecting minimal residual disease or isolated tumour cells; therefore, the use of more sensitive methods, such as immunocytochemistry or molecular approaches, should be considered in future studies to improve the detection of minimal tumour cell dissemination. In addition, the clinical and procedural heterogeneity of the study population, including the inclusion of different colorectal pathologies and surgical procedures, may represent a further limitation and may have affected internal validity. Another important limitation is the absence of a control group in which the specimen was extracted without the use of a retrieval bag, which prevents direct comparison with alternative extraction strategies and precludes direct evidence of its protective effect, thereby further limiting causal inference. Finally, given the exploratory and hypothesis-generating nature of the study, no a priori sample size calculation or predefined target effect size was performed and no formal correction for multiple testing was applied; therefore, the risk of both type I and type II error cannot be excluded, and the inferential strength of the observed associations is limited.

Despite these limitations, the study offers several important strengths, including its prospective multicentre design and the systematic collection of three intraoperative samples at pre-defined stages of the operation, providing a unique perspective on the temporal dynamics of bacterial contamination during minimally invasive colorectal surgery. The systematic use of a retrieval bag enabled the safe assessment of intra-bag contamination, and the combined microbiological and cytological analysis add further evidence regarding the oncological safety of these procedures. Overall, the findings indicate that bacterial contamination progressively increases throughout surgery, with the highest intra-abdominal risk occurring after vascular ligation and bowel division, and that contamination peaks again during specimen extraction, although effectively contained by the retrieval bag. The absence of tumour cell detection further reinforces that adherence to oncological principles, particularly the no-touch technique, is effective in preventing malignant cell dissemination.

In conclusion, the retrieval bag demonstrated a potential protective effect by containing bacterial spillage, with cytology confirming an extremely low risk of tumour cell dissemination within the limits of the present study. Given the sample size, particularly for cytological assessment, these findings should be interpreted with caution, and future validation studies on larger, adequately powered cohorts are warranted to better define the impact of specimen extraction techniques on oncological and infectious outcomes. In this context, well-designed randomized controlled trials comparing different specimen extraction strategies would be essential to establish causal relationships and confirm the protective role of retrieval bags. Despite these limitations, the results of the present study strongly support the routine consideration of retrieval bags in minimally invasive colorectal surgery. Their use should be seriously contemplated not only in right hemicolectomies, but also in left-sided resections and anterior resection of the rectum, where the risk of contamination during specimen extraction may be even more pronounced.

## Figures and Tables

**Table 1:** Patients’ demographics.

	Patients N = 88
**Gender ratio (man/woman), n (%)**	55 (62.5):33 (37.5)
**Mean age ± standard deviation age, years**	65.4 ± 11.5
**Mean Body Mass Index ± standard deviation, kg/m^2^**	26.7 ± 4.2
**Comorbidities, n (%)** - Hypertension - Diabetes mellitus - Ischemic heart disease - Smoke habits - Dyslipidemia - Ischemic heart disease - Hypothyroidism - Chronic obstructive pulmonary disease - Obstructive sleep apnea - Atrial fibrillation - Cardiac arrhythmia - Chronic renal insufficiency - Familial polyposis	31 (35.2) 17 (19.3) 41 (10.3) 9 (10.2) 6 (6.8) 3 (3.4) 3 (3.4) 3 (3.4) 3 (3.4) 2 (2.3) 1 (1.1) 1 (1.1) 1 (1.1)
**American Society of Anesthesiologists grade** - I - II - III	15 (17) 42 (47.7) 31 (35.2)
**Previous abdominal surgery, n (%)**	31 (35.2)
- Cholecystectomy - Appendectomy - Inguinal hernia repair - Hysterectomy - Prostatectomy - Caesarean section - Ovariectomy - Nephrectomy - Left hemicolectomy for cancer - Right hemicolectomy for cancer - Umbilical hernia repair	8 (9.1) 5 (5.7) 4 (4.5) 3 (3.4) 2 (2.3) 2 (2.3) 2 (2.3) 2 (2.3) 1 (1.1) 1 (1.1) 1 (1.1)
**Indication to surgery, n (%)** - Adenocarcinoma - Giant polyps with high-grade dysplasia - Chronic diverticulitis	51 (58) 18 (20.5) 19 (21.6)
**Lesion site, n (%)** - Right colon - Sigmoid colon - Rectum - Left colon - Recto-sigmoid junction - Transverse colon	33 (37.5) 29 (33) 11 (12.5) 6 (6.8) 5 (5.7) 4 (4.5)

**Table 2:** Perioperative results.

	Patients N = 88
**Approach, n (%)** - Single port - Conventional laparoscopy	49 (55.7) 39 (44.3)
**Surgical procedure, n (%)** - Right hemicolectomy - Sigmoidectomy - Anterior resection of the rectum - Left hemicolectomy	36 (40.9) 23 (26.1) 16 (18.2) 13 (14.8)
**Intraoperative complications, n (%)** - Bleeding - Positive hydropneumatics test	3 (3.4) 1 (1.1)
**Conversion to open surgery, n (%)**	-
**Type of mechanical anastomosis, n (%)** - Intracorporeal side-to-side - Intracorporeal end-to-end - Extracorporeal side-to-side - Extracorporeal side-to-end - Colostomy	25 (28.4) 25 (28.4) 20 (22.7) 17 (19.3) 1 (1.1)
**Mean operative time ± standard deviation, minutes**	128.1 ± 39.2
**Complications, n**	
**(%, Clavien-Dindo classification grade)**	24 (27.3)
- Wound infection - Postoperative ileus - Pneumonia - Bleeding - Anastomotic leakage	8 (1.8, II) 2 (3.3, I) 2 (2.3, II) 2 (2.3, III-b) 10 (11.4, III-b)
**Mean hospital stay ± standard deviation, days**	8.4 ± 5.9
**Mortality, n (%)**	-
**Definitive histology, n (%)** - Diverticular disease - pT0N0 - pTisN0 - pT1N0 - pT2N0 - pT2N1 - pT3N0 - pT3N1 - pT3N2 - pT4N1 - pT4N2	22 (25) 5 (5.7) 8 (9.1) 3 (3.4) 10 (11.4) 2 (2.3) 20 (22.7) 11 (12.5) 4 (4.5) 1 (1.1) 2 (2.3)
**Mean number of harvested lymph nodes ± standard deviation**	15.8 ± 6.1
**R0 resection, n (%)**	-
**Recurrence, n (%)**	-

**Table 3:** Microbiological agent retrieved in each sample.

Microbiological Agent	Number of Patients (%)
**First Sample Positive After Pneumoperitoneum Induction and Abdominal Exploration: 10 (11.4%)**	
*Escherichia coli*	7 (8)
*Enterococcus*	2 (2.3)
*Bacteroides vulgatus*	1 (1.1)
Mixed flora	1 (1.1)
Total	11 (12.5)
**Second sample positive after vascular and bowel division: 33 (37.5%)**	
*Escherichia coli*	16 (18.2)
Mixed flora	13 (14.8)
*Enterococcus faecium*	5 (5.7)
*Clostridium*	2 (2.3)
*Bacillus megaterium*	1 (1.1)
Bacteroides	1 (1.1)
*Klebsiella pneumoniae*	1 (1.1)
*Bacteroides vulgatus*	1 (1.1)
*Lactobacillus*	1 (1.1)
*Morganella morganii*	1 (1.1)
*Prevotella denticola*	1 (1.1)
*Candida albicans*	1 (1.1)
*Aeromonas hydrophila*	1 (1.1)
Total	45 (51.1)
**Third sample positive after extraction of the resected bowel: 59 (67%)**	
*Escherichia coli*	26 (29.5)
Mixed flora	14 (15.9)
Bacteroides (diastasonis, fragilis, thetaiotaomicron, vulgatus)	11 (12.5)
*Enterococcus*	6 (6.8)
Streptococcus (salivarius, constellatus, gallolyticus)	5 (5.7)
*Bacteroides vulgatus*	3 (3.4)
*Pseudomonas aeruginosa*	3 (3.4)
*Serratia marcescens*	3 (3.4)
*Citrobacter*	2 (2.3)
*Morganella morganii*	2 (2.3)
*Candida*	1 (1.1)
*Enterobacter*	1 (1.1)
*Klebsiella pneumoniae*	1 (1.1)
*Parabacteroides*	1 (1.1)
*Staphylococcus aureus*	1 (1.1)
Total	80 (90.1)

**Table 4:** Factors associated with intraoperative bacterial contamination at different surgical stages.

Variable	*p* Value Sample 1	*p* Value Sample 2	*p* Value Sample 3	Statistical Test
**Age**	0.245	0.393	0.889	T-student
**Gender**	0.167	0.776	0.178	Chi-square
**Body mass index**	0.295	0.803	0.570	T-student
**ASA grade**	0.704	0.851	0.503	Chi-square
**Approach**	0.502	0.013 *	0.327	Chi-square
**Intracorporeal anastomosis**	0.945	0.022 *	0.945	Chi-square
**Wound infection**	1.000	0.309	1.000	Chi-square
**Anastomotic leakage**	0.275	0.739	0.291	Chi-square
**Malignant disease**	0.213	0.038 *	0.487	Chi-square
**Hospital stay**	0.014 *	<0.001 *	0.559	Mann–Whitney U

ASA: American Society of Anesthesiologists. * Statistically significant differences in bold.

## Data Availability

The original contributions presented in this study are included in the article. Further inquiries can be directed to the corresponding author.
